# Contemporary fluid management, humidity, and patent ductus arteriosus management strategy for premature infants among 336 hospitals in Asia

**DOI:** 10.3389/fped.2024.1336299

**Published:** 2024-02-29

**Authors:** Yao-Chi Hsieh, Mei-Jy Jeng, Ming-Chih Lin, Yuh-Jyh Lin, Rinawati Rohsiswatmo, Rizalya Dewi, Seok Chiong Chee, Siew Hong Neoh, Belen Amparo E. Velasco, Ma. Lourdes S. Imperial, Pracha Nuntnarumit, Sopapan Ngerncham, Yun Sil Chang, Sae Yun Kim, Bin Huey Quek, Zubair Amin, Satoshi Kusuda, Fuyu Miyake, Tetsuya Isayama

**Affiliations:** ^1^ Children’s Medical Center, Taichung Veterans General Hospital, Taichung, Taiwan; ^2^ Institute of Emergency and Critical Care Medicine, School of Medicine, National Yang Ming Chiao Tung University, Taipei, Taiwan; ^3^ Department of Pediatrics, Taipei Veterans General Hospital, Taipei, Taiwan; ^4^ Department of Pediatrics, School of Medicine, National Yang Ming Chiao Tung University, Taipei, Taiwan; ^5^ Department of Post-Baccalaureate Medicine, College of Medicine, National Chung Hsing University, Taichung, Taiwan; ^6^ Department of Food and Nutrition, Providence University, Taichung, Taiwan; ^7^ School of Medicine, Chung Shan Medical University, Taichung, Taiwan; ^8^ Department of Pediatrics, National Cheng-Kung University Hospital, Tainan, Taiwan; ^9^ Department of Child Health, Faculty of Medicine Universitas Indonesia, Cipto Mangunkusumo General Hospital, Jakarta, Indonesia; ^10^ Budhi Mulia Women and Children Hospital, Pekanbaru, Indonesia; ^11^ School of Medicine, Faculty of Health and Medical Sciences, Taylor’s University, Subang Jaya, Malaysia; ^12^ Philippine Children’s Medical Center, Manila, Philippines; ^13^ Dr. Jose Fabella Memorial Hospital, Manila, Philippines; ^14^ Department of Pediatrics, Faculty of Medicine Ramathibodi Hospital, Mahidol University, Bangkok, Thailand; ^15^ Department of Pediatrics, Faculty of Medicine Siriraj Hospital, Mahidol University, Bangkok, Thailand; ^16^ Department of Pediatrics, Samsung Medical Center, Sungkyunkwan University School of Medicine, Seoul, Republic of Korea; ^17^ Department of Pediatrics, College of Medicine, The Catholic University of Korea, Seoul, Republic of Korea; ^18^ Department of Neonatology, KK Women’s and Children’s Hospital, Singapore, Singapore; ^19^ Department of Neonatology, Khoo Teck Puat-National University Children's Medical Institute, National University Hospital, Singapore, Singapore; ^20^ Neonatal Research Network of Japan, Tokyo, Japan; ^21^ Department of Pediatrics, Kyorin University, Mitaka, Tokyo, Japan; ^22^ Division of Neonatology, National Center for Child Health and Development, Tokyo, Japan

**Keywords:** prematurity, patent ductus arteriosus, fluid management, humidity, AsianNeo

## Abstract

**Objectives:**

The management of patent ductus arteriosus (PDA) is a critical concern in premature infants, and different hospitals may have varying treatment policies, fluid management strategies, and incubator humidity. The Asian Neonatal Network Collaboration (AsianNeo) collected data on prematurity care details from hospitals across Asian countries. The aim of this study was to provide a survey of the current practices in the management of PDA in premature infants in Asian countries.

**Methods:**

AsianNeo performed a cross-sectional international questionnaire survey in 2022 to assess the human and physical resources of hospitals and clinical management of very preterm infants. The survey covered various aspects of hospitals resources and clinical management, and data were collected from 337 hospitals across Asia. The data collected were used to compare hospitals resources and clinical management of preterm infants between areas and economic status.

**Results:**

The policy of PDA management for preterm infants varied across Asian countries in AsianNeo. Hospitals in Northeast Asia were more likely to perform PDA ligation (*p *< 0.001) than hospitals in Southeast Asia. Hospitals in Northeast Asia had stricter fluid restrictions in the first 24 h after birth for infants born at <29 weeks gestation (*p *< 0.001) and on day 14 after birth for infants born at <29 weeks gestation (*p *< 0.001) compared to hospitals in Southeast Asia. Hospitals in Northeast Asia also had a more humidified environment for infants born between 24 weeks gestation and 25 weeks gestation in the first 72 h after birth (*p *< 0.001). A logistic regression model predicted that hospitals were more likely to perform PDA ligation for PDA when the hospitals had a stricter fluid planning on day 14 after birth [Odds ratio (OR) of 1.70, *p* = 0.048], more incubator humidity settings (<80% vs. 80%–89%, OR of 3.35, *p *= 0.012 and <80% vs. 90%–100%, OR of 5.31, *p* < 0.001).

**Conclusions:**

In advanced economies and Northeast Asia, neonatologists tend to adopt a more conservative approach towards fluid management, maintain higher incubator humidity settings and inclined to perform surgical ligation for PDA.

## Introduction

During fetal development, the ductus arteriosus shunts blood from the pulmonary artery to the aorta. After birth, the ductus arteriosus should gradually close. When it remains open after birth, it is known as a patent ductus arteriosus (PDA) (1, 2). PDA can occur due to various factors, including prematurity, respiratory distress syndrome, neonatal asphyxia, and congenital heart disease. PDA is especially important in extremely low birth weight (ELBW) premature infants (3, 4) as changes in hemodynamics can lead to complications such as intraventricular hemorrhage (IVH), acute kidney injury (AKI), bronchopulmonary dysplasia (BPD), pulmonary hemorrhage, and mortality (5–14). There are several methods for PDA treatment, including conservative treatment, pharmacological treatment, and surgical ligation (3–5, 7, 15–22). However, there is currently no consensus on the optimal treatment for PDA as different experts, such as neonatologists and cardiologists, have different views. Moreover, there are significant variations in PDA management among different countries, regions, and hospitals (5, 16, 23–26). Conservative treatment, such as fluid restriction, is one way to manage PDA (27–31), but evaluating fluid status in premature infants is challenging, and fluid management strategies and incubator humidity settings vary among hospitals.

Asian Neonatal Network Collaboration (AsianNeo) is an international collaboration between neonatal networks in Asia. It aims to evaluate and compare perinatal and neonatal medical systems and clinical practices, as well as the outcomes of sick newborn infants, particularly those born with very low birth weight or very prematurely, in order to improve the quality of care provided to these vulnerable infants (32).

The aim of this study was to provide a survey of the current practices in the management of PDA in premature infants in Asian countries and to improve the overall quality of care for this vulnerable population.

## Materials and methods

### AsianNeo

Asian Neonatal Network Collaboration is a collaborative effort involving neonatal networks from eight countries: Indonesia, Japan, Malaysia, Philippines, Singapore, South Korea, Taiwan, and Thailand. Led by Dr. Tetsuya Isayama from the National Center for Child Health and Development in Tokyo, Japan, AsianNeo includes members from various fields, such as neonatologists, pediatricians, healthcare professionals, researchers, policy makers, and patients and their families. The goal of AsianNeo is to improve the quality of care and outcomes of prematurity by evaluating and comparing the perinatal and neonatal medical systems as well as clinical practices in Asian countries through surveys, clinical research, and quality improvement activities.

### Data source

The AsianNeo conducted a cross-sectional, international questionnaire survey at the institutional level to assess the human and physical resources of hospitals and clinical management of very preterm infants in 2022. The survey was distributed to participating hospitals through the representatives of each neonatal network. The questionnaire was written in English and coded into the Survey Monkey® online tool (33), and responses were collected in English or translated into native languages for non-English-speaking countries. The questionnaire covered various aspects of hospitals resources and clinical management, such as Neonatal Intensive Care Units (NICU) level, patient volume, circulation, nutrition/feeding, neurology, follow-up systems, and more. The data collected were used to compare hospitals resources and clinical management of preterm infants between the participating countries or regions. The focus of our investigation was the physical resources of hospitals, clinical management of PDA, prematurity fluid management, and incubator humidity settings.

### Data ownership, intellectual properties, and ethics approval

The AsianNeo Bureau, located at the National Center for Child Health and Development (NCCHD) in Tokyo, Japan, oversaw the research program. Policies and procedures for data transfer, usage, analysis, and publication were established through discussion among the steering committee and agreements signed between each network and the AsianNeo Bureau. The research ethics board of the NCCHD has approved this study protocol (ID 2020-244), and data transfer agreements were signed by all networks prior to transferring data. The AsianNeo Bureau only received aggregate data and did not collect any patient-identifiable information. Each network was responsible for obtaining regulatory or research ethics approval for the de-identified data transfer to the AsianNeo Bureau at the NCCHD. This study was approved by the Institutional Review Board (IRB) of Taichung Veterans General Hospital. (Approval number: CE23034B).

### Statistical analysis

This study collected 337 questionnaires, and 1 questionnaire was information uncompleted, then be excluded. Thus we total analyzed 336 hospitals in Asia, divided into Northeast Asian countries (including Korea and Japan) and Southeast Asian countries (including Malaysia, Philippines, Singapore, Taiwan, Thailand, and Indonesia). We also use economic development level for region differentiation. According to the International Monetary Fund (IMF) classifies the world into advanced economies and developing/emerging economies based on three primary criteria: (1) per capita income level, (2) export diversification, and (3) degree of integration into the global financial system (34). According to IMF, we categorize Japan, South Korea, Taiwan, and Singapore as advanced economies. Indonesia, Thailand, Malaysia, and the Philippines are classified as emerging and developing economies (35).

The AsianNeo Surveys questionnaire was utilized, which was completed by hospitals representatives. Using chi-square tests, the characteristics of each hospitals were analyzed, including the number of NICU beds, the availability of a fellowship program, affiliation with a medical university and so on. Subsequently, a chi-square test was used to analyze whether there were any differences in PDA management between the two regions. Additionally, differences in fluid management and incubator setting of humidity policies were analyzed between Southeast Asian and Northeast Asian countries using chi-square tests.

To further investigate the associations of PDA treatment policy with fluid management and humidity setting of incubators, chi-square tests were performed. Then, a logistic regression model was used to evaluate the relationships of fluid management with humidity of the incubator and whether PDA ligation was favored for the hospitals. A *p*-value <0.05 was considered statistically significant. All analyses were conducted using the SAS statistical package (SAS System for Windows, Version 9.4, SAS Institute Inc., Cary, NC, USA).

## Results

### Characteristics of hospitals

In this study, we analyzed the differences between hospitals in Northeast Asia and Southeast Asia, and we found many differences between the two groups. First, the number of Level 2 NICU beds in Northeast Asia was significantly lower than that in Southeast Asia (*p *< 0.001, Table 1). The proportion of hospitals in Northeast Asia offering fellowships was higher than that in Southeast Asia (68.92% vs. 29.27%, *p *< 0.001, Table 1). In a comparison of infants requiring level 3 care per year, most of the hospitals in Northeast Asia had 100–199 and 200–299 infants, while most of those in Southeast Asia had >400 infants (*p *< 0.001, Table 1). Comparing very low-birth-weight infants per year (birth weight <1,500 g), most of the hospitals in Northeast Asia had 20–39 and 40–74 infants, while most of those in Southeast Asia had >75 infants. With respect to extremely low-birth-weight infants (birth weight <1,000 g) per year, most of the hospitals in Northeast Asia had 0–19 infants, while most of those in Southeast Asia had 0–19 and 20–39 infants. Regarding deliveries in hospital per year, most of the hospitals in Northeast Asia had 500–999 infants, while most of those in Southeast Asia had >1,500 infants (*p* < 0.001, Table 1). The proportions of outborn infants <29 weeks gestational age were mostly <1% and 1%–9% in Northeast Asia, but mostly 1%–9% and 10%–49% in Southeast Asia (*p *< 0.001, Table 1). In terms of whether the hospital provided cardiac surgery for neonates (PDA clipping, etc.), the proportion was higher in Northeast Asia than in Southeast Asia (63.32% vs. 50.31%, *p *< 0.001, Table 1). Similarly, in terms of whether the hospital provided ROP treatment (laser, anti-VEGF injection, etc.), the proportion was higher in Northeast Asia than in Southeast Asia (94.59% vs. 68.67%, *p *< 0.001, Table 1). In terms of categorizing hospitals into advanced economies hospitals and emerging and developing economies hospitals, detailed information on the characteristics of hospitals can be found in Supplementary Appendix Table S2.

**Table 1:** Characteristics of hospitals.

	Southeast Asia hospitals	Northeast Asian hospitals	*P*-value
	Number	Number	
Number of Level-3 NICU beds^a^			
0–9	65 (36.2%)	55 (37.2%)	0.60
10–14	47 (28.3%)	38 (25.7%)	
15–19	24 (14.5%)	30 (20.3%)	
>20	30 (18.1%)	25 (16.9%)	
Number of Level-2 NICU beds^b^			
5–9	39 (24.5%)	27 (18.8%)	<0.001
10–14	36 (22.6%)	33 (22.9%)	
15–19	18 (11.3%)	40 (27.8%)	
20–24	18 (11.3%)	25 (17.4%)	
>25	48 (30.2%)	19 (13.2%)	
Fellowship program			
Hospitals did not offer Fellowship program training	116 (70.7%)	46 (31.1%)	<0.001
Hospitals offer Fellowship program training	48 (29.3%)	102 (68.9%)	
Medical university			
Hospitals not affiliated with medical university	68 (41.7%)	74 (50.0%)	0.14
Hospitals affiliated with a medical university	95 (58.3%)	74 (50.0%)	
Infants requiring level 3 care per year			
<50	25 (15.2%)	20 (13.6%)	<0.001
50–99	18 (10.9%)	18 (12.2%)	
100–199	34 (20.6%)	48 (32.7%)	
200–299	21 (12.7%)	32 (21.8%)	
300–399	19 (11.5%)	15 (10.2%)	
>400	48 (29.1%)	14 (9.5%)	
Very low-birth-weight infants (birth weight <1,500 g) per year			
0–19	24 (14.6%)	31 (21.1%)	<0.001
20–39	34 (20.6%)	47 (32.0%)	
40–74	40 (24.2%)	56 (38.1%)	
>75	67 (40.6%)	13 (8.8%)	
Extremely low-birth-weight infants (birth weight <1,000 g) per year			
0–19	76 (46.1%)	87 (61.3%)	<0.001
20–39	50 (30.3%)	42 (29.6%)	
40–74	28 (17.0%)	13 (9.2%)	
>75	11 (6.7%)	0 (0.0%)	
Deliveries in hospital per year			
<500	9 (5.5%)	41 (28.3%)	<0.001
500–999	27 (16.5%)	72 (49.7%)	
1,000–1,499	24 (14.6%)	24 (16.6%)	
>1,500	104 (63.4%)	8 (5.5%)	
Proportion of outborn infants <29 weeks gestational age			
<1%	37 (22.4%)	77 (53.1%)	<0.001
1%–9%	61 (37.0%)	50 (34.5%)	
10%–49%	48 (29.1%)	12 (8.3%)	
50%–89%	11 (6.7%)	1 (0.7%)	
90%–100%	8 (4.9%)	5 (3.5%)	
Services available in hospital			
Cardiac surgery of neonates (PDA clipping, etc.)			
Not available	80 (49.7%)	55 (37.7%)	0.034
Available	81 (50.3%)	91 (62.3%)	
Gastrointestinal surgery of neonates (laparotomy, etc.)			
Not available	34 (20.6%)	29 (19.6%)	0.82
Available	131 (79.4%)	119 (80.4%)	
ROP treatment (laser, anti-VEGF injection, etc.)			
Not available	52 (31.3%)	8 (5.4%)	<0.001
Available	114 (68.7%)	140 (94.6%)	
Neurosurgery (shunt surgery for hydrocephaly, etc.)			
Not available	41 (24.7%)	42 (28.4%)	0.46
Available	125 (75.3%)	106 (71.6%)	

^a^ Level-3 NICU: having ability of taking care of infants born at <32 weeks gestations or with birth weight <1,500 g, those with critical illness, or those on advanced respiratory support (intermittent mandatory ventilation, HFO, NIPPV, or CPAP).

^b^ Level-2 NICU: having the ability to taking care of infants born at ≥32 weeks gestation or with birth weight ≥1,500 g without advanced respiratory support who need mild support for their immaturity or transitional illness (those on supplemental oxygen or gastric tubing). Level 2 units may take care of those on advanced respiratory support only for short period [e.g., <24 h].

### PDA management and attitude of fluid and humidity for preterm infants

**Figure 1:**
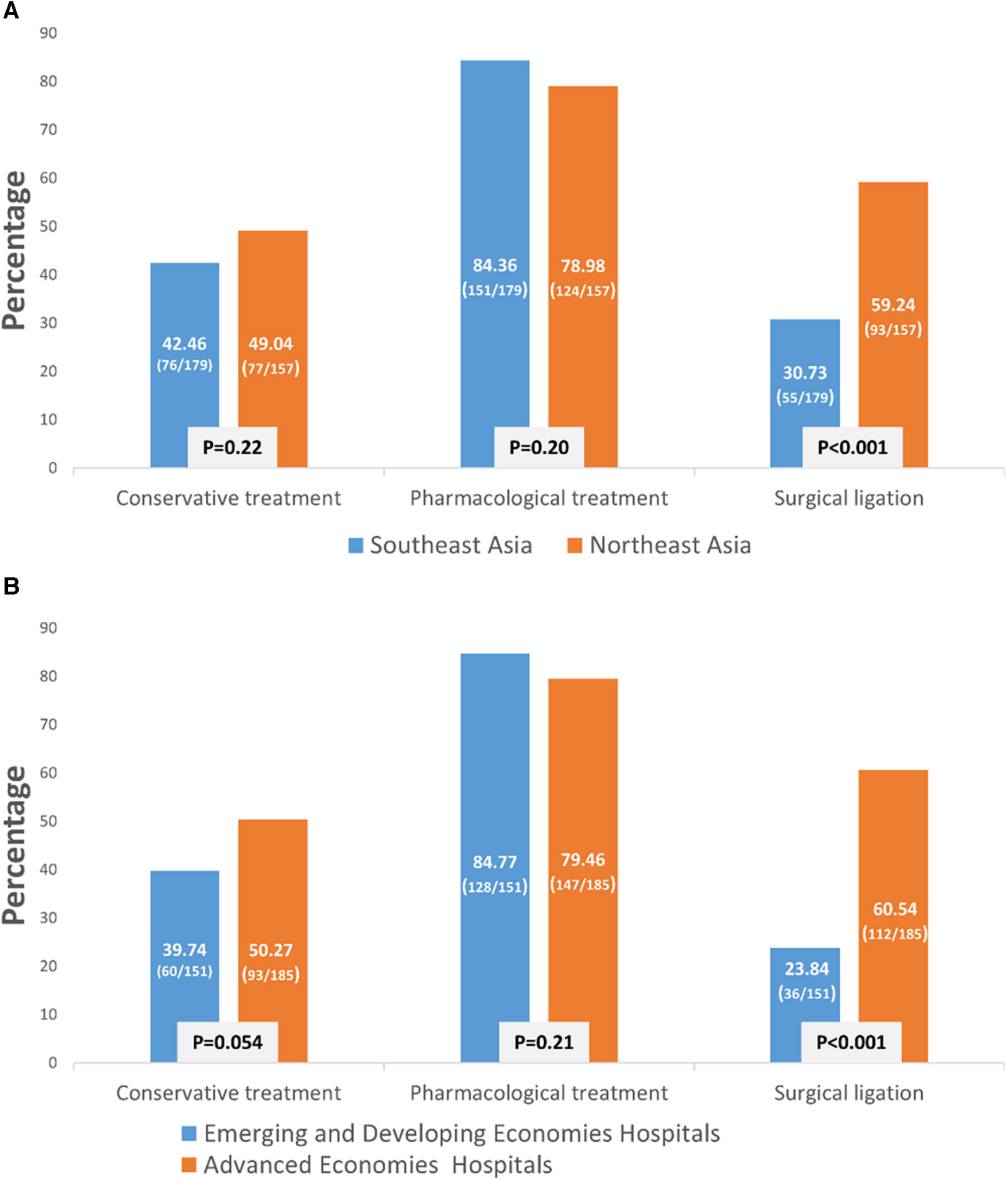
(**A**) PDA management strategy in East Asia. Neonatologists of Northeast Asia tend to ligate PDA more. (**B**) Neonatologists of advanced economies hospitals tend to ligate PDA than emerging and developing economies hospitals.

**Figure 2:**
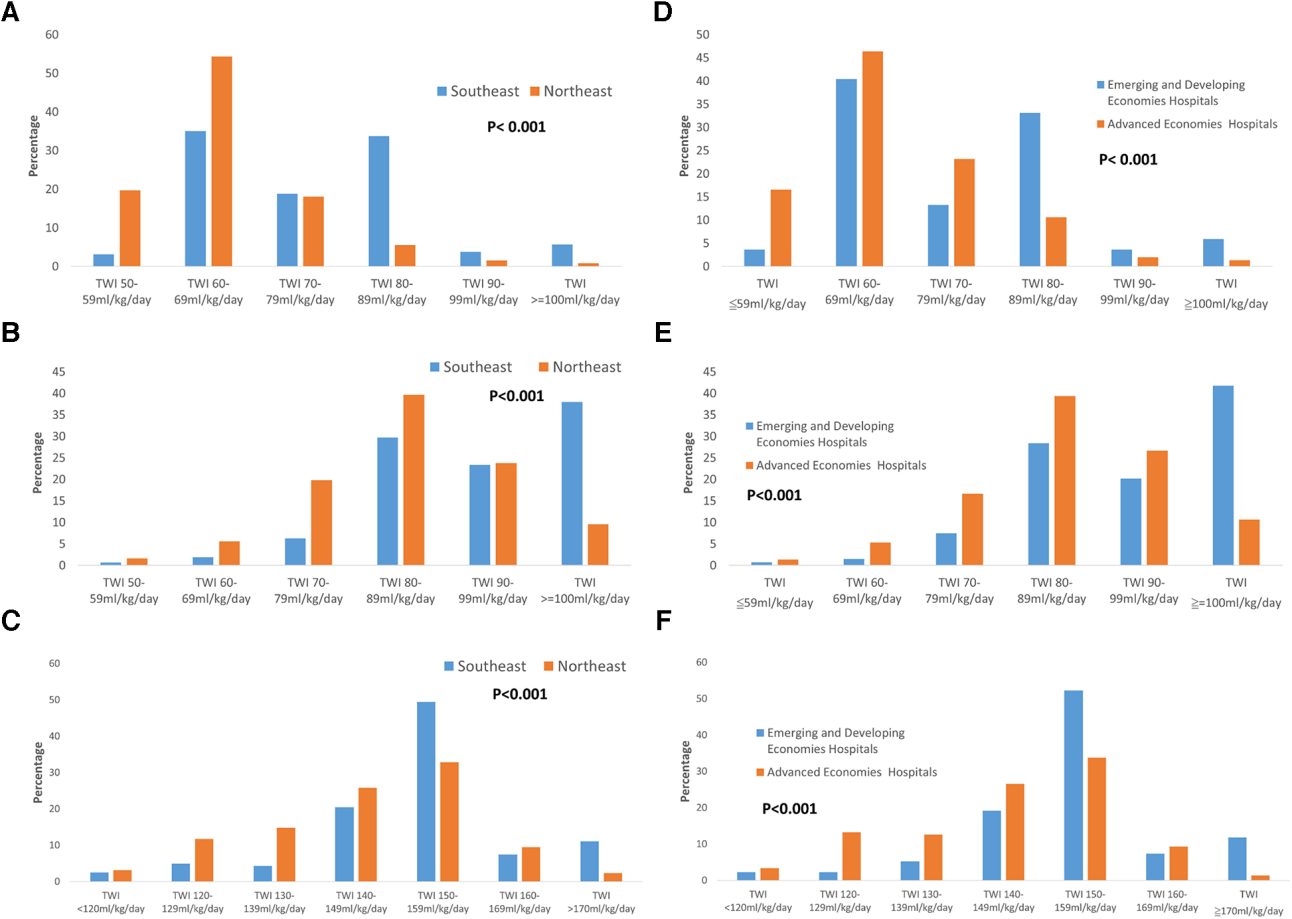
(**A**) Fluid planning in the first 24 h after birth in infants born at <29 weeks’ gestation. Hospitals in Northeast Asia had stricter fluid restrictions compared to hospitals in Southeast Asia *TWI, total water intake. (**B**) Fluid planning in 48–72 h after birth in infants born at <29 weeks’ gestation. Hospitals in Northeast Asia had stricter fluid restrictions compared to hospitals in Southeast Asia *TWI, total water intake. (**C**) Fluid planning on 14 days after birth in infants born at <29 weeks’ gestation. Hospitals in Northeast Asia had stricter fluid restrictions compared to hospitals in Southeast Asia *TWI, total water intake. (**D**) Fluid planning in the first 24 h after birth in infants born at <29 weeks’ gestation. Advanced economies hospitals had stricter fluid restrictions compared to emerging and developing economies hospitals *TWI, total water intake. (**E**) Fluid planning in 48–72 h after birth in infants born at <29 weeks’ gestation. Advanced economies hospitals had stricter fluid restrictions compared to emerging and developing economies hospitals *TWI, total water intake. (**F**) Fluid planning on 14 days after birth in infants born at <29 weeks’ gestation. Advanced economies hospitals had stricter fluid restrictions compared to emerging and developing economies hospitals *TWI, total water intake.

**Figure 3:**
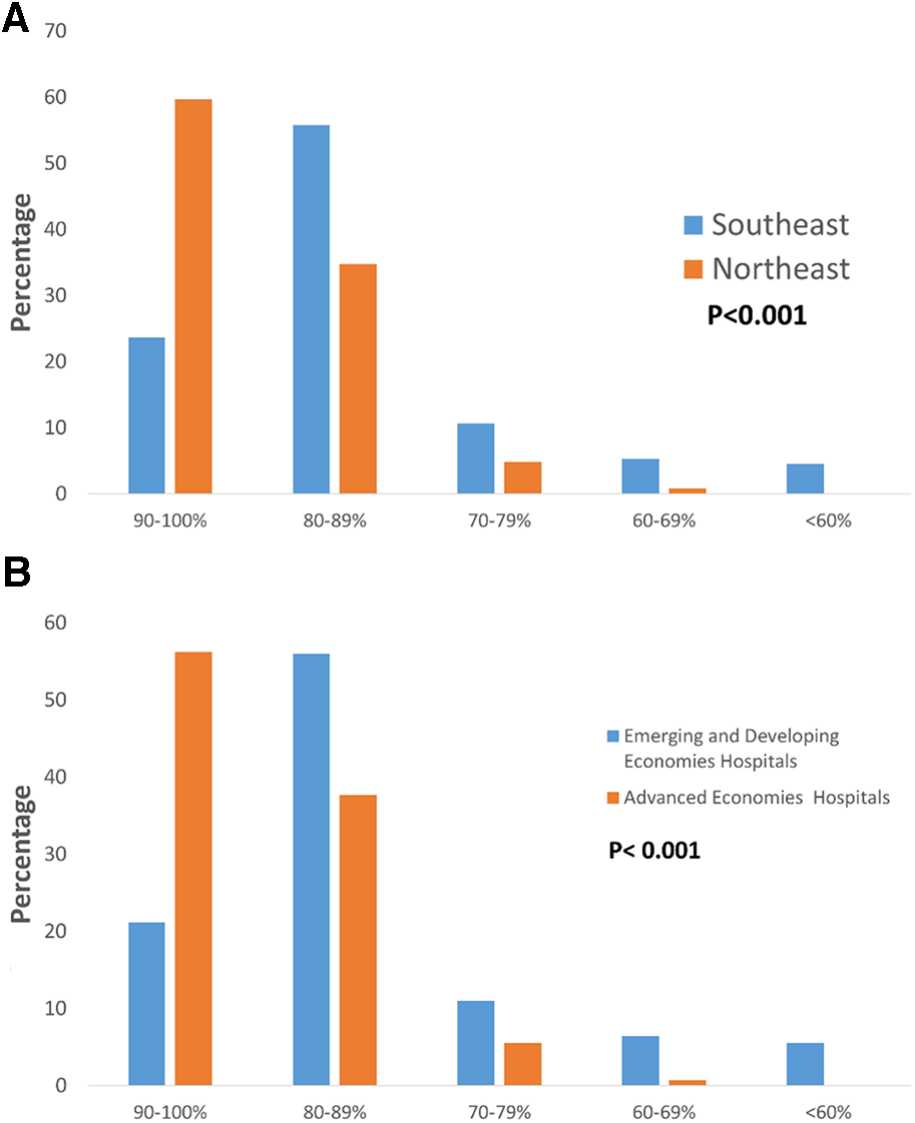
(**A**) Humidity setting of incubators for infants born at 24–25 weeks’ gestation in the first 72 h after birth. Neonatologists of Northeast Asia tend to set higher humidity. (**B**) Humidity setting of incubators for infants born at 24–25 weeks’ gestation in the first 72 h after birth. Neonatologists of advanced economies hospitals tend to set higher humidity.

The policy of PDA management for preterm infants varied between Northeast Asia and Southeast Asia. Hospitals in Northeast Asia were more inclined to perform PDA ligation than hospitals in Southeast Asia (Figure 1A, *p *< 0.001). Similarly, to previous finding, advanced economies hospitals were also more likely to performed PDA ligation than emerging and developing economies hospitals. Additionally, hospitals in Northeast Asia had stricter fluid restrictions in the first 24 h after birth for infants born at <29 weeks' gestation compared to hospitals in Southeast Asia (Figure 2A, *p *< 0.001). Moreover, hospitals in Northeast Asia imposed stricter fluid restrictions for infants born at <29 weeks' gestation during the 48–72 h after birth in comparison to hospitals in Southeast Asia (Figure 2B, *p *< 0.001). Similarly, hospitals in Northeast Asia had stricter fluid restrictions for infants born at <29 weeks' gestation on day 14 after birth as opposed to hospitals in Southeast Asia (Figure 2C, *p *< 0.001). In comparing advanced economies to emerging and developing economies, hospitals in advanced economies displayed consistently stricter fluid management practices. This was evident within the first 24 h after birth for infants born at <29 weeks gestation (Figure 2D, *p* < 0.001), between 48 and 72 h post-birth (Figure 2E, *p* < 0.001), and on day 14 after birth (Figure 2F, *p* < 0.001). Furthermore, hospitals in Northeast Asia provided a more humidified environment for infants born between 24 weeks gestation and 25 weeks’ gestation in the first 72 h after birth (Figure 3A, *p *< 0.001). The advanced economies hospitals also had more humidity incubator setting than emerging and developing economies hospitals (Figure 3B, *p* < 0.001). These findings underscore the disparities in PDA management and fluid restriction practices across Asian countries in AsianNeo.

### Associations between fluid management, humidity, and PDA management strategy

In our further analysis of the management of PDA, the water supply plan and humidity in the hospitals, we found that the more aggressively the PDA was treated, the more likely the hospitals was to limit water intake and use a more humidified incubator. We further analyzed the data and found that hospitals with a total water intake (TWI) plan of less than 79 ml per kilogram per day in the first 24 h after birth were more likely to receive PDA ligation than those with TWI more than 79 ml per kilogram per day (Table 2, *p *= 0.007). Similarly, hospitals with a TWI plan of less than 139 ml per kilogram per day on day 14 after birth were more likely to receive PDA ligation than those with TWI more than 139 ml per kilogram per day (Table 2, *p *= 0.024). In addition, hospitals with more humidifier incubator settings were more likely to receive PDA ligation (Table 2, *p *< 0.001).

**Table 2:** Associations between fluid management, humidity, and PDA ligation strategy.

	Hospitals not using surgical ligation for PDA	Hospitals using surgical ligation for PDA	*P*-value
1. Fluid planning in the first 24 h after birth in infants born at <29 weeks gestation			
TWI ≦ 79 ml/kg/day	92 (65.3%)	116 (79.5%)	0.007
TWI > 79 ml/kg/day	49 (34.8%)	30 (20.6%)	
2. Fluid planning on 14 days after birth in infants born at <29 weeks gestation			
TWI ≦ 149 ml/kg/day	45 (33.3%)	70 (48.6%)	0.009
TWI > 149 ml/kg/day	90 (66.7%)	74 (51.4%)	
3. Humidity setting of incubators for infants born at 24–25 weeks gestation in the first 72 h after birth on average			
<80%	27 (22.5%)	7 (5.2%)	<0.001
80–89%	56 (46.7%)	60 (44.4%)	
90–100%	37 (30.8%)	68 (50.4%)	

TWI, total water intake; PDA, patent ductus arteriosus.

**Figure 4:**
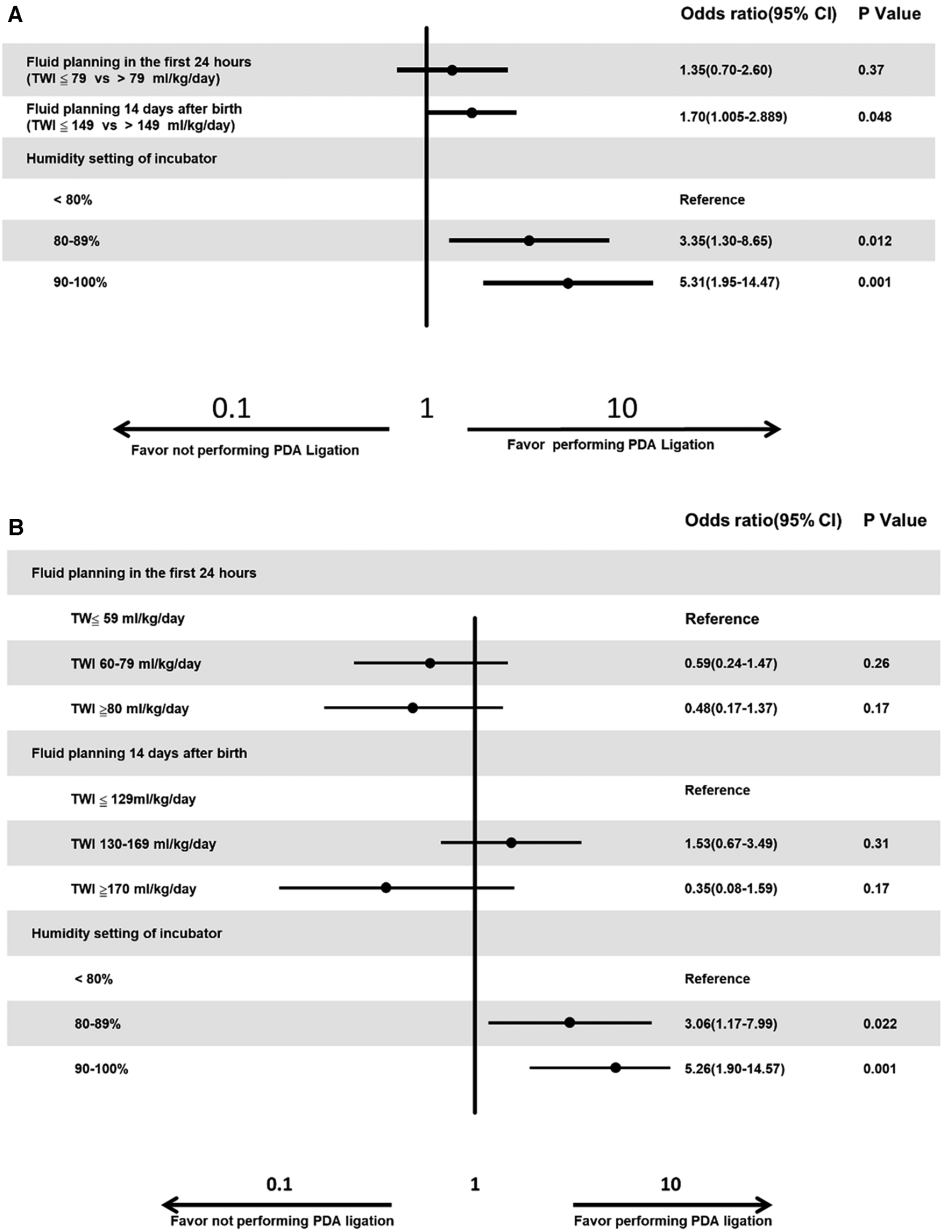
(**A**) Multiple logistic regression analysis for the factors that influence the tendency of PDA ligation. Neonatologists more likely to perform PDA ligation if they have stricter fluid management or higher humidity setting. (**B**) Multiple logistic regression analysis for the factors that influence the tendency of PDA ligation. Neonatologists more likely to perform PDA ligation if they have higher humidity setting.

A logistic regression model predicted that hospitals were more likely to perform PDA ligation for PDA when the hospitals had a stricter fluid planning on day 14 after birth [Odds ratio (OR) of 1.70, *p *= 0.048], more incubator humidity settings (<80% vs. 80%–89%, OR of 3.35, *p *= 0.012 and <80% vs. 90%–100%, OR of 5.31, *p *< 0.001, Figure 4A). We also categorized fluid management into three groups. For the initial 24 h after birth, we defined TW ≤59 ml/kg/day as conservative, TWI 60–79 ml/kg/day as reasonable, and TWI ≥80 ml/kg/day as excessive. Similarly, for the 14-day-old group, we categorized TWI ≤129 ml/kg/day as conservative, TWI 130–169 ml/kg/day as reasonable, and TWI ≥170 ml/kg/day as excessive. Our logistic regression model revealed a higher likelihood of hospitals performing PDA ligation when their incubator humidity settings were lower (<80% vs. 80%–89%, OR of 3.06, *p* = 0.022, and <80% vs. 90%–100%, OR of 5.26, *p* = 0.001, Figure 4B).

## Discussion

This study was a large-scale research project conducted across Asia. Hospitals in Northeast Asia are more likely to perform PDA surgical ligation, in comparison to hospitals in Southeast Asia. Moreover, hospitals in Northeast Asia implemented stricter fluid restrictions, providing reduced fluid intake to preterm infants, whereas hospitals in Southeast Asia had more lenient fluid management practices. Interestingly, hospitals in Northeast Asia were observed to have higher humidity settings in their incubators when compared to those in Southeast Asia.

For premature infants undergoing conservative treatment for patent ductus arteriosus (PDA), water restriction is a commonly used method (27–31). However, there is no consensus on the amount of water needed to maintain the infant's nutrition and needs while also treating PDA. Fluid assessment of premature infants has always been a challenging and crucial issue. Various methods, including body weight change, urine output, urine specific gravity, blood pressure, and echocardiography, can be used to assess the fluid status of premature infants. Nonetheless, there is no unified view among experts on the appropriate amount of water to administer (5, 16, 23–26).

In premature infants born at 22–24 weeks gestation, it is recommended to provide a starting fluid intake of 100–170 ml/kg/day when using a humidified incubator (36). For infants weighing less than 750 g at birth, a recommended fluid intake of 100–140 ml/kg/day is advised on the first day (37). For infants weighing between 750 and 1,000 g at birth, a recommended fluid intake of 100–120 ml/kg/day is advised on the first day (37). However, in our study, we found that the majority of Asian hospitals had a total water intake (TWI) of 60–69 ml/kg/day in the first 24 h after birth for infants born at less than 29 weeks gestation. These hospitals accounted for 43.5% of the total hospitals surveyed. This suggests that hospitals in Asia tend to have more stringent fluid restriction policies.

For premature infants weighing less than 1,000 g, a fluid intake of 140–160 ml/kg/day is recommended on the seventh day and beyond (37, 38). In our study, we examined the fluid management practices in Asian hospitals for premature infants born at less than 29 weeks gestation at 14 days after birth. The majority of hospitals (42.06%) implemented a total water intake (TWI) of 150–159 ml/kg/day during this period. These findings suggest that as the patient's condition stabilizes, the daily fluid intake gradually increases, and the recommended fluid intake for Asian hospitals is consistent with other regions.

A full-term baby's skin is fully keratinized, but premature babies' skin is different. The epidermis of premature infants gradually matures from 23 weeks to around 32 weeks (39, 40). This fragile skin cannot provide a complete barrier to prevent water loss, and premature babies have a larger surface-to-body weight ratio than adults (41, 42). Therefore, premature infants are prone to losing a large amount of water and body temperature through their skin, leading to dehydration and hypothermia. We found that in Asian hospitals, which had premature babies born at 24–25 weeks, the humidity settings in the incubator during the first 72 h were mostly set at 90%–100% (39.60%) and 80%–89% (43.70%). Other studies have shown that using humidifiers in incubators to maintain humidity levels above 85% can effectively reduce water loss from the premature infant's immature skin during the first week after birth (43, 44). However, excessively high humidity levels can lead to the proliferation of microorganisms and pathogens, resulting in infections (45). Research indicates that using incubator humidity levels of 60%–70% can effectively mature the skin and reduce long-term water loss (45). Therefore, there is currently no consensus on the optimal incubator humidity settings in different regions.

In our study, hospitals tended to opt for PDA ligation when they maintained stricter fluid management by day 14 after birth and had more incubator humidity settings (Figure 4A). However, after categorizing fluid management into three groups—conservative, reasonable, and excessive—the significance of fluid planning on day 14 after birth diminished (Figure 4B). This change might be attributed to the majority of cases falling within the TWI 130–169 ml/kg/day category, which was not further subdivided compared to the division at 149 ml/kg/day.

There are several risk factors associated with PDA in premature infants, including respiratory distress syndrome (RDS), high volume of intravenous fluids (>170 ml/kg per day) in the first week, sepsis, prolonged rupture of membranes, furosemide use, male sex (18), and aminoglycoside antibiotics (46). Conversely, the use of antenatal corticosteroids (47) and maternal hypertension (48) has been shown to decrease the incidence of PDA. The optimal timing for surgical PDA ligation in premature infants remains a topic of controversy (49). Generally, surgical intervention is considered when PDA causes severe hemodynamic changes or leads to clinical symptoms such as respiratory compromise (e.g., requiring persistent mechanical support), heart failure, hypotension, oliguria, wide pulse pressure (18, 50, 51), or evidence of a large left-to-right ductus shunt on cardiac sonography (e.g., large left atrial-to-aortic root diameter ratio, large diameter of the ductus arteriosus) (51, 52). These indications prompt the consideration of surgical PDA ligation in premature infants. However, the potential impact of the higher humidity settings in incubators and stricter fluid administration in the early stages of premature infants on the development of significant hemodynamic changes leading to surgical PDA ligation remains uncertain. Further research is needed to investigate the relationship between these factors and the occurrence of hemodynamic compromise that may necessitate surgical intervention for PDA in premature infants.

In our study, we observed a correlation between hospitals employing more conservative fluid management and a higher likelihood of conducting PDA surgical ligation. We hypothesize that this finding may stem from hospitals prioritizing or being more cautious about the potential adverse effects of PDA. Consequently, they might initiate restricted fluid administration as an initial conservative treatment strategy for preterm infants even before PDA progresses to a hemodynamically significant stage or before considering PDA surgical ligation.

This study has several limitations. Firstly, not all hospitals in Asian countries were included in the study, which may have introduced selection bias, thereby limiting the generalizability of the findings. Secondly, Japan and South Korea were categorized as Northeast Asia, while Indonesia, Malaysia, Philippines, Singapore, Taiwan, and Thailand were classified as Southeast Asia based on geographical location. However, when considering preterm infants' care, categorizing countries based on factors like economic development and survival rates might be more meaningful. Unfortunately, this study did not include data on economic development, survival rates, or similar indicators for these countries. Thirdly, the absence of clear indications for PDA treatment, absence of the steps involved in PDA treatment, and the lack of individual center's outcome data, including survival rate, rates of NEC, BPD, and ROP, have constrained our ability to evaluate whether diverse management strategies influence outcomes of those premature infants. Additionally, designating a single physician per hospital to respond to the questionnaire might not accurately represent the hospital's practices, as different physicians within the same facility may adopt diverse approaches. Moreover, the questionnaire's extensive and diverse content made it challenging to complete. Furthermore, the questionnaire's validity and reliability, as utilized in this study, have not been formally validated. The questionnaire has not been linked to individual center's outcome data. Further research is warranted to determine if conservative fluid management and higher incubator settings contribute to better PDA treatment outcome. These limitations highlight the need for further research and collaboration to address these challenges and provide more comprehensive insights into the management of patent ductus arteriosus in premature infants.

## Conclusion

In advanced economies and Northeast Asia, neonatologists tend to adopt a more conservative approach towards fluid management, maintain higher incubator humidity settings and inclined to perform surgical ligation for PDA.

## Data Availability

The data presented in this article are not readily available because data release is not allowed by AsianNeo. Requests to access the datasets should be directed to Dr. Fuyu Miyake/ fmiyake1228@gmail.com.

## Supplementary material

Supplementary Appendix Table S1 Number of Level-3 NICUs and Level-2 NICUs in each country.

Supplementary Appendix Table S2 Characteristics of hospitals.

## References

[1] Hamrick SE Hansmann G. Patent ductus arteriosus of the preterm infant. Pediatrics. (2010) 125(5):1020–30. 10.1542/peds.2009-3506 20421261

[2] Hundscheid T van den Broek M van der Lee R de Boode WP. Understanding the pathobiology in patent ductus arteriosus in prematurity-beyond prostaglandins and oxygen. Pediatr Res. (2019) 86(1):28–38. 10.1038/s41390-019-0387-7 30965358

[3] Lee JA Kim MJ Oh S Choi BM. Current status of therapeutic strategies for patent ductus arteriosus in very-low-birth-weight infants in Korea. J Korean Med Sci. (2015) 30 Suppl 1(Suppl 1):S59–66. 10.3346/jkms.2015.30.S1.S59 26566359 PMC4641065

[4] Su BH Lin HY Chiu HY Tsai ML Chen YT Lu IC. Therapeutic strategy of patent ductus arteriosus in extremely preterm infants. Pediatr Neonatol. (2020) 61(2):133–41. 10.1016/j.pedneo.2019.10.002 31740267

[5] Benitz WE, Committee on F, Newborn AAoP. Patent ductus arteriosus in preterm infants. Pediatrics. (2016) 137(1):e20153730. 10.1542/peds.2015-3730 26672023

[6] Brown ER. Increased risk of bronchopulmonary dysplasia in infants with patent ductus arteriosus. J Pediatr. (1979) 95(5 Pt 2):865–6. 10.1016/S0022-3476(79)80454-0 490263

[7] Conrad C Newberry D. Understanding the pathophysiology, implications, and treatment options of patent ductus arteriosus in the neonatal population. Adv Neonatal Care. (2019) 19(3):179–87. 10.1097/ANC.0000000000000590 30720481

[8] Cunha GS Mezzacappa-Filho F Ribeiro JD. Risk factors for bronchopulmonary dysplasia in very low birth weight newborns treated with mechanical ventilation in the first week of life. J Trop Pediatr. (2005) 51(6):334–40. 10.1093/tropej/fmi051 15927945

[9] Dollberg S Lusky A Reichman B. Patent ductus arteriosus, indomethacin and necrotizing enterocolitis in very low birth weight infants: a population-based study. J Pediatr Gastroenterol Nutr. (2005) 40(2):184–8. 10.1097/00005176-200502000-00019 15699694

[10] Garland J Buck R Weinberg M. Pulmonary hemorrhage risk in infants with a clinically diagnosed patent ductus arteriosus: a retrospective cohort study. Pediatrics. (1994) 94(5):719–23. 10.1542/peds.94.5.719 7936902

[11] Lipman B Serwer GA Brazy JE. Abnormal cerebral hemodynamics in preterm infants with patent ductus arteriosus. Pediatrics. (1982) 69(6):778–81. 10.1542/peds.69.6.778 7079043

[12] Muehlbacher T Bassler D Bryant MB. Evidence for the management of bronchopulmonary dysplasia in very preterm infants. Children. (2021) 8(4):298. 10.3390/children8040298 33924638 PMC8069828

[13] Puthiyachirakkal MA. Pathophysiology and management of intraventricular hemorrhage in preterm infants. EC Paediatrics. (2018) 7(6):537–45.

[14] Su BH Lin HY Huang FK Tsai ML Huang YT. Circulatory management focusing on preventing intraventricular hemorrhage and pulmonary hemorrhage in preterm infants. Pediatr Neonatol. (2016) 57(6):453–62. 10.1016/j.pedneo.2016.01.001 26993561

[15] El-Khuffash A Weisz DE McNamara PJ. Reflections of the changes in patent ductus arteriosus management during the last 10 years. Arch Dis Child Fetal Neonatal Ed. (2016) 101(5):F474–8. 10.1136/archdischild-2014-306214 27118761

[16] Bose CL Laughon M. Treatment to prevent patency of the ductus arteriosus: beneficial or harmful? J Pediatr. (2006) 148(6):713–4. 10.1016/j.jpeds.2006.03.015 16769371

[17] de Waal K Prasad R Kluckow M. Patent ductus arteriosus management and the drift towards therapeutic nihilism—what is the evidence? Semin Fetal Neonatal Med. (2021) 26(2):101219. 10.1016/j.siny.2021.101219 33653600

[18] Gillam-Krakauer M Reese J. Diagnosis and management of patent ductus arteriosus. Neoreviews. (2018) 19(7):e394–402. 10.1542/neo.19-7-e394 30505242 PMC6269146

[19] Hermes-DeSantis ER Clyman RI. Patent ductus arteriosus: pathophysiology and management. J Perinatol. (2006) 26 Suppl 1(1):S14–8, discussion S22–3. 10.1038/sj.jp.7211465 16625216

[20] Hundscheid T Onland W Kooi EMW Vijlbrief DC de Vries WB Dijkman KP Expectant management or early ibuprofen for patent ductus arteriosus. N Engl J Med. (2023) 388(11):980–90. 10.1056/NEJMoa2207418 36477458

[21] Mitra S Florez ID Tamayo ME Aune D Mbuagbaw L Veroniki AA Effectiveness and safety of treatments used for the management of patent ductus arteriosus (PDA) in preterm infants: a protocol for a systematic review and network meta-analysis. BMJ Open. (2016) 6(7):e011271. 10.1136/bmjopen-2016-011271 27456327 PMC4964163

[22] Sankar MN Bhombal S Benitz WE. PDA: to treat or not to treat. Congenit Heart Dis. (2019) 14(1):46–51. 10.1111/chd.12708 30811796

[23] Benitz WE. Treatment of persistent patent ductus arteriosus in preterm infants: time to accept the null hypothesis? J Perinatol. (2010) 30(4):241–52. 10.1038/jp.2010.3 20182439

[24] Benitz WE. Hey, doctor, leave the PDA alone. Pediatrics. (2017) 140(2):e20170566. 10.1542/peds.2017-0566 28701391

[25] Hundscheid T El-Khuffash A McNamara PJ de Boode WP. Survey highlighting the lack of consensus on diagnosis and treatment of patent ductus arteriosus in prematurity. Eur J Pediatr. (2022) 181(6):2459–68. 10.1007/s00431-022-04441-8 35305143 PMC9110525

[26] Sathanandam S Whiting S Cunningham J Zurakowski D Apalodimas L Waller BR Practice variation in the management of patent ductus arteriosus in extremely low birth weight infants in the United States: survey results among cardiologists and neonatologists. Congenit Heart Dis. (2019) 14(1):6–14. 10.1111/chd.12729 30811803

[27] Dudley S Sen S Hanson A El Khuffash A Levy PT. The role of furosemide and fluid management for a hemodynamically significant patent ductus arteriosus in premature infants. J Perinatol. (2022) 42(12):1703–7. 10.1038/s41372-022-01450-1 35840707

[28] Francescato G Capolupo I Cerbo RM Doni D Ficial B Fiocchi S Fluid restriction in management of patent ductus arteriosus in Italy: a nationwide survey. Eur J Pediatr. (2023) 182(1):393–401. 10.1007/s00431-022-04685-4 36374300

[29] Liu C Shi Y. Association between fluid balance and treatment outcome of ibuprofen for patent ductus arteriosus in preterm infants. Rev Cardiovasc Med. (2023) 24(3):78. 10.31083/j.rcm2403078 PMC11263992 39077496

[30] Sung SI Chang YS Ahn SY Jo HS Yang M Park WS. Conservative non-intervention approach for hemodynamically significant patent ductus arteriosus in extremely preterm infants. Front Pediatr. (2020) 8:605134. 10.3389/fped.2020.605134 33425816 PMC7786118

[31] Lalitha R Surak A Bitar E Hyderi A Kumaran K. Fluid and electrolyte management in preterm infants with patent ductus arteriosus. J Neonatal Perinatal Med. (2022) 15(4):689–97. 10.3233/NPM-210943 35599502

[32] Asian Neonatal Network Collaboration. Available online at: https://asian-neo.org/index.html (accessed June 6, 2023).

[33] Momentive Inc. C, USA. SurveyMonkey. Available online at: https://www.surveymonkey.com/ (accessed June 6, 2023).

[34] World Economic Outlook. Frequently Asked Questions. [updated Oct 10, 2023]. (2023). Available online at: https://www.imf.org/en/Publications/WEO/frequently-asked-questions (accessed December 20, 2023).

[35] World Economic and Financial Surveys, World Economic Outlook. Database—WEO Groups and Aggregates Information. [updated October 2021]. (2023). Available online at: https://www.imf.org/external/pubs/ft/weo/2021/02/weodata/groups.htm (accessed December 20, 2023).

[36] Agren J Segar JL Soderstrom F Bell EF. Fluid management considerations in extremely preterm infants born at 22–24 weeks of gestation. Semin Perinatol. (2022) 46(1):151541. 10.1016/j.semperi.2021.151541 34848064

[37] Christine A Gleason MD. Avery’s Diseases of the Newborn. 10th ed. Philadelphia: Elsevier (2018). p. 374–5.

[38] Chawla D Agarwal R Deorari AK Paul VK. Fluid and electrolyte management in term and preterm neonates. Indian J Pediatr. (2008) 75(3):255–9. 10.1007/s12098-008-0055-0 18376094

[39] Hoath SB Narendran V. Adhesives and emollients in the preterm infant. Semin Neonatol. (2000) 5(4):289–96. 10.1053/siny.2000.0015 11032712

[40] Rutter N. Applied physiology: the newborn skin. Curr Paediatr. (2003) 13(3):226–30. 10.1016/S0957-5839(03)00008-3

[41] Chow JM Douglas D. Fluid and electrolyte management in the premature infant. Neonatal Netw. (2008) 27(6):379–86. 10.1891/0730-0832.27.6.379 19065967

[42] Fidler HL. Incubator humidity: more than just something to sweat about!! Adv Neonatal Care. (2011) 11(3):197–9. 10.1097/ANC.0b013e31821d0074 21730914

[43] Agren J Sjors G Sedin G. Ambient humidity influences the rate of skin barrier maturation in extremely preterm infants. J Pediatr. (2006) 148(5):613–7. 10.1016/j.jpeds.2005.11.027 16737871

[44] Antonucci R Porcella A Fanos V. The infant incubator in the neonatal intensive care unit: unresolved issues and future developments. J Perinat Med. (2009) 37(6):587–98. 10.1515/JPM.2009.109 19591569

[45] Glass L Valdez A. Preterm infant incubator humidity levels: a systematic review. Adv Neonatal Care. (2021) 21(4):297–307. 10.1097/ANC.0000000000000791 33009156

[46] Vucovich MM Cotton RB Shelton EL Goettel JA Ehinger NJ Poole SD Aminoglycoside-mediated relaxation of the ductus arteriosus in sepsis-associated PDA. Am J Physiol Heart Circ Physiol. (2014) 307(5):H732–40. 10.1152/ajpheart.00838.2013 24993047 PMC4187398

[47] Chorne N Jegatheesan P Lin E Shi R Clyman RI. Risk factors for persistent ductus arteriosus patency during indomethacin treatment. J Pediatr. (2007) 151(6):629–34. 10.1016/j.jpeds.2007.05.007 18035143

[48] Koch J Hensley G Roy L Brown S Ramaciotti C Rosenfeld CR. Prevalence of spontaneous closure of the ductus arteriosus in neonates at a birth weight of 1,000 g or less. Pediatrics. (2006) 117(4):1113–21. 10.1542/peds.2005-1528 16585305

[49] Noori S McCoy M Friedlich P Bright B Gottipati V Seri I Failure of ductus arteriosus closure is associated with increased mortality in preterm infants. Pediatrics. (2009) 123(1):e138–44. 10.1542/peds.2008-2418 19117835

[50] Chiruvolu A Punjwani P Ramaciotti C. Clinical and echocardiographic diagnosis of patent ductus arteriosus in premature neonates. Early Hum Dev. (2009) 85(3):147–9. 10.1016/j.earlhumdev.2008.12.008 19217224

[51] Afiune JY Singer JM Leone CR. Echocardiographic post-neonatal progress of preterm neonates with patent ductus arteriosus. J Pediatr (Rio J). (2005) 81(6):454–60. 10.2223/JPED.1419 16385362

[52] Braulio R Gelape CL Araujo FD Brandao KN Abreu LD Costa PH Indicators of surgical treatment of patent ductus arteriosus in preterm neonates in the first week of life. Rev Bras Cir Cardiovasc. (2013) 28(4):504–8. 10.5935/1678-9741.20130082 24598956 PMC4389417

