# Supplementary material for: Contemporary fluid management, humidity, and patent ductus arteriosus management strategy for premature infants among 336 hospitals in Asia
Source: Front Pediatr. 2024 Feb 29;12:1336299. doi: 10.3389/fped.2024.1336299 (PMC10937448; doi:10.3389/fped.2024.1336299)
Supplement: Supplementary Appendix Table S1 — Number of Level-3 NICUs and Level-2 NICUs in each country. [file Table1.docx]

# Supplementary Material

| [**Appendix**](https://www.google.com/search?sca_esv=589727162&sxsrf=AM9HkKl47YMaYwY5NvcWGeI4xCv7aXjS9Q:1702285729225&q=appendix&spell=1&sa=X&ved=2ahUKEwj1_8jBhIeDAxWYavUHHb5dAvgQkeECKAB6BAgFEAI) **Table 1.** Number of Level-3 NICUs and Level-2 NICUs in each country | | | | | | | |  |  |
| --- | --- | --- | --- | --- | --- | --- | --- | --- | --- |
|  | Indonesia | Japan | South  Korean | Malaysia | Philippines | Singapore | Taiwan | Thailand | Total |
| Number of Level-3 NICUs | 38 | 144 | 13 | 35 | 16 | 3 | 25 | 62 | 336 |
| Number of Level-2 NICUs | 38 | 144 | 13 | 35 | 16 | 3 | 25 | 62 | 336 |
