# Supplementary material for: Contemporary fluid management, humidity, and patent ductus arteriosus management strategy for premature infants among 336 hospitals in Asia
Source: Front Pediatr. 2024 Feb 29;12:1336299. doi: 10.3389/fped.2024.1336299 (PMC10937448; doi:10.3389/fped.2024.1336299)
Supplement: Supplementary Appendix Table S2 — Characteristics of hospitals. [file Table2.docx]

[**Appendix**](https://www.google.com/search?sca_esv=589727162&sxsrf=AM9HkKl47YMaYwY5NvcWGeI4xCv7aXjS9Q:1702285729225&q=appendix&spell=1&sa=X&ved=2ahUKEwj1_8jBhIeDAxWYavUHHb5dAvgQkeECKAB6BAgFEAI) **Table 2.** Characteristics of hospitals

|  |  | |  |  | |  |
| --- | --- | --- | --- | --- | --- | --- |
|  | Emerging and Developing Economies Hospitals | Advanced Economies Hospitals | | |  | |
|  | **Number** | **Number** | | | **P value** | |
| Number of Level-3 NICU beds* |  |  | | | 0.51 | |
| 0~9 | 58(41.1%) | 62(35.8%) | | |  | |
| 10~14 | 40(28.4%) | 45(26.0%) | | |  | |
| 15~19 | 20(14.2%) | 34(19.7%) | | |  | |
| >20 | 23(16.3%) | 32(18.5%) | | |  | |
| Number of Level-2 NICU beds** |  |  | | | <0.001 | |
| 5~9 | 38(27.9%) | 28(16.8%) | | |  | |
| 10~14 | 32(23.5%) | 37(22.2%) | | |  | |
| 15~19 | 12(8.8%) | 46(27.5%) | | |  | |
| 20~24 | 14(10.3%) | 29(17.4%) | | |  | |
| >25 | 40(29.4%) | 27(16.2%) | | |  | |
|  |  |  | | |  | |
| Fellowship program |  |  | | |  | |
| Hospital did not offer Fellowship program training | 110(78.6%) | 52(30.2%) | | | <0.001 | |
| Hospital offer Fellowship program training | 30(21.4%) | 120(69.8%) | | |  | |
| Medical university |  |  | | | 0.65 | |
| Hospital not affiliated with medical university. | 61(44.2%) | 81(46.8%) | | |  | |
| Hospital affiliated with a medical university. | 77(55.8%) | 92(53.2%) | | |  | |
| Infants requiring level 3 care per year |  |  | | | 0.005 | |
| <50 | 22(15.7%) | 23(13.4%) | | |  | |
| 50~99 | 14(10.0%) | 22(12.8%) | | |  | |
| 100~199 | 30(21.4%) | 52(30.2%) | | |  | |
| 200~299 | 18(12.9%) | 35(20.4%) | | |  | |
| 300~399 | 15(10.7%) | 19(11.1%) | | |  | |
| >400 | 41(29.3%) | 21(12.2%) | | |  | |
| Very low-birth-weight infants (birth weight < 1500g) per year |  |  | | | <0.001 | |
| 0-19 | 17(12.1%) | 38(22.2%) | | |  | |
| 20-39 | 30(21.3%) | 51(29.8%) | | |  | |
| 40-74 | 32(22.7%) | 64(37.4%) | | |  | |
| >75 | 62(44.0%) | 18(10.5%) | | |  | |
| Extremely low-birth-weight infants (birth weight < 1000g) per year |  |  | | | <0.001 | |
| 0-19 | 63(44.7%) | 100(60.2%) | | |  | |
| 20-39 | 41(29.1%) | 51(30.7%) | | |  | |
| 40-74 | 27(19.2%) | 14(8.4%) | | |  | |
| >75 | 10(7.1%) | 1(0.6%) | | |  | |
| Deliveries in hospital per year |  |  | | | <0.001 | |
| <500 | 6(4.3%) | 44(26.0%) | | |  | |
| 500~999 | 20(14.3%) | 79(46.8%) | | |  | |
| 1000~1499 | 20(14.3%) | 28(16.6%) | | |  | |
| >1500 | 94(67.1%) | 18(10.7%) | | |  | |
| Proportion of outborn infants < 29 weeks gestational age |  |  | | | <0.001 | |
| <1% | 32(22.9%) | 82(48.2%) | | |  | |
| 1~9% | 48(34.3%) | 63(37.1%) | | |  | |
| 10~49% | 43(30.7%) | 17(10.0%) | | |  | |
| 50~89% | 9(6.4%) | 3(1.8%) | | |  | |
| 90~100% | 8(5.7%) | 5(2.9%) | | |  | |
| Services available in hospital |  |  | | |  | |
| Cardiac surgery of neonates (PDA clipping, etc.) |  |  | | | <0.001 | |
| Not available | 78(56.9%) | 57(33.5%) | | |  | |
| Available | 59(43.1%) | 113(66.5%) | | |  | |
| Gastrointestinal surgery of neonates (laparotomy, etc.) |  |  | | | 0.17 | |
| Not available | 33(23.6%) | 30(17.3%) | | |  | |
| Available | 107(76.4%) | 143(82.7%) | | |  | |
| ROP treatment (laser, anti-VEGF injection, etc.) |  |  | | | <0.001 | |
| Not available | 51(36.2%) | 9(5.2%) | | |  | |
| Available | 90(63.8%) | 164(94.8%) | | |  | |
| Neurosurgery (shunt surgery for hydrocephaly, etc.) |  |  | | | 0.66 | |
| Not available | 39(27.7%) | 44(25.4%) | | |  | |
| Available | 102(72.3%) | 129(74.6%) | | |  | |
|  |  |  | | |  | |

*Level-3 NICU: having ability of taking care of infants born at < 32 weeks gestations or with birth weight < 1500g, those with critical illness, or those on advanced respiratory support (intermittent mandatory ventilation, HFO, NIPPV, or CPAP)

**Level-2 NICU: having the ability to taking care of infants born at ≥32 weeks gestation or with birth weight ≥ 1500g without advanced respiratory support who need mild support for their immaturity or transitional illness (those on supplemental oxygen or gastric tubing). Level 2 units may take care of those on advanced respiratory support only for short period [e.g. <24 hours].
